# Algae-Enhanced Electrospun Polyacrylonitrile Nanofibrous Membrane for High-Performance Short-Chain PFAS Remediation from Water

**DOI:** 10.3390/nano13192646

**Published:** 2023-09-26

**Authors:** Shobha Mantripragada, Dongyang Deng, Lifeng Zhang

**Affiliations:** 1 Department of Nanoengineering, Joint School of Nanoscience and Nanoengineering, North Carolina A&T State University, Greensboro, NC 27401, USA; 2 Department of Built Environment, College of Science and Technology, North Carolina A&T State University, Greensboro, NC 27411, USA

**Keywords:** electrospinning, polyacrylonitrile, algae, GenX, short-chain PFASs, water treatment

## Abstract

As a short-chain PFAS (per- and polyfluoroalkyl substance), GenX was produced in recent years to replace traditional long-chain PFASs, such as perfluorooctanoic acid (PFOA). However, GenX turns out to be more toxic than people originally thought, posing health risks as a persistent environmental pollutant. In this research, for the first time, we incorporated chlorella, a single-celled green freshwater microalga that grows worldwide, with polyacrylonitrile (PAN) in equal amounts in electrospun nanofibers and studied the capability of the electrospun PAN/Algae bicomponent nanofibrous membrane (ES(PAN/Algae)) to bind and remove GenX from water. The incorporation of algae demonstrated a synergistic effect and significantly improved the GenX removal efficiency of the nanofibrous membrane. The maximum GenX removal capacity reached 0.9 mmol/g at pH 6, which is significantly higher than that of most of the reported GenX adsorbents as well as activated carbon. The GenX removal mechanism was investigated and discussed by using water contact angle, zeta potential, FTIR, and XPS techniques. This research demonstrated the potential to make highly efficient adsorbent/filter materials from common and economic materials to practically remediate short-chain PFASs from various water bodies.

## 1. Introduction

Per- and polyfluoroalkyl substances (PFASs) are synthetic chemicals that were first introduced in the 1940s and have been used in a variety of applications such as firefighting foam, stain-resistant carpet, and non-stick and stain-resistant products, etc. These substances have become emerging water contaminants due to their extremely stable C-F bond and water solubility. PFASs are listed as persistent organic pollutants (POPs) under the Stockholm Convention due to their persistence, toxicity, and bioaccumulation in the ecosystem [1]. Exposure to these substances has raised serious health concerns both in animals and humans such as liver damage, kidney cancer, etc., as well as developmental effects affecting unborn children [2]. Due to their adverse health effects, the two most widely used and studied PFASs, perfluorooctanoic acid (PFOA) and perfluorooctane sulfonate (PFOS), have been replaced by shorter chain counterparts with a popular one known as GenX (ammonium salt of hexafluoropropylene oxide dimer acid, IUPAC name: ammonium 2,3,3,3-terafluoro-2-(1,1,2,2,3,3,3-heptafluropropoxy) propanoate), which has been produced since 2010. The distribution of GenX has been assessed in multiple environmental studies across multiple domains including air, rain, surface water, and groundwater, as well as drinking water, around the world [3,4]. Research has demonstrated that GenX is equally, if not more, toxic than long-chain PFAS counterparts and has also raised big health concerns [5]. In 2022, the United States Environmental Protection Agency announced a stringent lifetime health advisory for GenX at 10 ng/L. However, there is only limited research regarding the remediation of GenX from water and there is an urgent need to develop adsorbent materials to effectively remove GenX from water [6].

To date, activated carbon as a popular commercial adsorbent for water contaminants has been used as a standard measure for PFAS remediation from water. However, studies have demonstrated that activated carbon would not be effective in remediating short-chain PFAS molecules, such as GenX, because they may break through activated carbon adsorbent within a shorter time frame than that of their long-chain counterparts [7]. Therefore, there is a growing demand for developing novel adsorbent materials to address effective GenX removal from water bodies. In past years, a few GenX adsorbent materials such as amine-functionalized covalent organic framework [8], dual-functionalized hydrogels [9], and ionic fluorogels [10] have been explored to remove GenX from water with weight-normalized GenX removal capacities comparable to that of activated carbon (~0.79 mmol/g) [11]. In our previous research, for the first time, we evaluated a popular filter/adsorbent material for water treatment, i.e., polyacrylonitrile (PAN), in the form of a nanofibrous membrane through electrospinning to remediate GenX from water [12]. By performing surface modification on an electrospun PAN nanofibrous membrane and converting nitrile groups to amidoxime functional groups, we promoted GenX removal capacity by 88% to ~0.6 mmol/g at pH 4, which is close and comparable to most of the other reported adsorbents along with activated carbon for GenX remediation from water. However, there is still room for improvement in GenX removal efficiency as well as material sustainability.

With the world-wide mission for sustainable development, integration of natural resources with adsorbent materials for GenX remediation from water becomes increasingly attractive. Algae have gained special attention as renewable bioresources because of their high photosynthetic efficiency and high growth rates. Algae contain bioactive components such as polysaccharides, proteins, lipids, vitamins, and minerals [13,14], and the functional groups in algae have exhibited great potential to remove heavy metals, dyes, polyaromatic compounds, and pharmaceutical compounds from wastewater [15,16]. Our recent research revealed that soy protein coating can significantly improve the GenX adsorption efficiency of cellulose-based nanofibers [17]. Considering the protein component in algae and the difficulty of electrospinning standalone algae, the incorporation of algae in PAN nanofibers through electrospinning could inject sustainability to PAN nanofibrous adsorbent/filter materials with much potential to enhance the effectiveness of GenX remediation from water.

In this research, we prepared PAN/Algae nanofibrous membranes from electrospinning and studied their capability to bind and adsorb GenX from water. The algae we used are chlorella, which are single-celled green microalgae that grow naturally in freshwater all over the world and have been used as a food supplement due to their high protein content [18]. The GenX removal mechanism was investigated by utilizing water contact angle, zeta potential, Fourier-transform infrared (FTIR) spectroscopy, and X-ray photon spectroscopy (XPS). By including 50 wt.% of chlorella algae in electrospun PAN nanofibers, the resultant nanofibrous membrane ES(PAN/Algae) showed an exciting GenX removal capacity of ~0.9 mmol/g. This research shed light on the development of effective adsorbent/filter materials from common and popular materials for practical short-chain PFAS remediation from water.

## 2. Materials and Methods

### 2.1. Materials

Polyacrylonitrile (PAN, MW = 150,000) and N, N-Dimethylformamide (DMF, 99%) were purchased from Sigma-Aldrich (St. Louis, MO, USA). Chlorella powder (algae) was purchased from Nuts.com. GenX was purchased from Synquest Laboratories. All materials were used as received without further processing. Type 1 Deionized (DI) water was used from a Millipore DI water system.

### 2.2. Preparation of Electrospun Nanofibers

*Chlorella* powder (the Algae) was weighed and finely dispersed into DMF solvent through 30 min sonication and then introduced into a PAN-DMF solution to make a DMF solution of PAN/Algae (50/50, wt./wt.) at a total concentration of 10 wt.%. The solution was continuously stirred for another 24 h to make a homogenous solution for electrospinning. The electrospinning solution was loaded into a 10 mL syringe that was connected to an 18 g blunt end needle. The electrospinning was carried out by applying 18 kV of positive voltage. The electrospun nanofibers were collected on a metal plate that was 15 cm away from the tip of the spinning needle and covered with aluminum foil. A PAN nanofibrous membrane was also prepared by electrospinning a 10 wt.% PAN-DMF solution; the membrane was used as the control. The as-spun fibers were placed in a fume hood to evaporate the remaining solvent and then kept in a desiccator for later use.

### 2.3. Preparation of GenX Water

The GenX water for adsorption tests was prepared by dissolving 100 mg of GenX in 1 L of Type 1 DI water. The pH of the solution was measured by using an Orion pH meter and adjusted by using diluted HCl or NaOH solution. We studied two pH values (pH 4 and 6) in this study.

### 2.4. Characterization

The surface morphology of the nanofibrous membrane was examined using a Zeiss Auriga FIB scanning electron microscope (SEM). Before analyzing, the SEM samples were sputter-coated with gold–palladium at a thickness of 8 nm to avoid surface charge accumulation. Elemental mapping was performed through an energy-dispersive X-ray spectrometer (EDX) that is equipped with the SEM. The average fiber diameter of respective nanofiber sample was obtained by measuring diameters of 30 randomly selected nanofibers in corresponding SEM images using Image J software (Version 1.53e). Chemical bonding in the nanofibrous membranes was characterized by attenuated total reflectance (ATR)-FTIR spectroscopy via an Agilent Varian 670 FTIR-ATR spectrometer using dry samples. Surface charge of the nanofibers was determined in DI water at pH values 4 and 6 using a Malvern Zeta Sizer ZEN3600 dynamic light scattering (DLS) instrument. Surface property of the nanofibrous membranes was assessed by water contact angle using a Rame Hart 260-F4 tensimeter. Surface elemental composition and bonding of the nanofibrous membranes were studied via an X-ray photon spectrometer (XPS, Thermo Scientific Escalab Xi+ (Waltham, MA, USA)). Avantage software (Version V5.9925) was used to process the data. The curve fitting of individual elements was conducted using a combination of Gaussian–Lorentz equations. All XPS spectra were charge-corrected by using C_1s_ binding energy of 284.8 eV.

### 2.5. GenX Sorption Test

Each GenX sorption test was carried out by using 100 mL of 100 mg/L GenX water at pH values 4 and 6. The GenX adsorption efficiency of all the nanofibrous membranes was accessed at room temperature using a filtration setup as reported in our previous research [19]. 0.024 g of the PAN/Algae bicomponent nanofibrous membrane as well as the PAN nanofibrous membrane as the control were weighed, respectively, and cut into circular shapes to seamlessly cover bottom of the filtering funnel as filtration media. The control filter medium of algae was prepared by placing 0.012 g of raw algae particles on top of 0.012 g of the PAN nanofibrous membrane that fitted into the bottom of the filtering funnel. The reason for preparing this control is because of the pass-through loss of raw algae particles during filtration through holes in the bottom of the filtering funnel when algae are directly applied.

Once the respective nanofibrous filter material was appropriately placed, 100 mL GenX water was poured into the funnel and allowed to flow through the nanofibrous filter via gravity. An amount of 10 mL of the filtered solution was placed into a polypropylene (PP) centrifuge tube and then subjected to centrifuge at 10,000 rpm for 30 min to remove possible nanofiber leftover in the solution. Then, 1 mL of the supernatant was taken and transferred to a quartz cuvette for GenX concentration measurement using a Varian Cary 6000i UV-Vis spectrometer with a wavelength set from 175 nm to 400 nm. A calibration curve was established based on the UV adsorption at wavelengths ranging from 189 to 190 nm with GenX concentrations ranging from 10 to 100 mg/L. A control UV absorbance of the respective filter membrane was acquired using DI water (without GenX) that went through the filtration setup under the same condition.

The percentage removal of GenX was calculated using Equation (1):

(1) $$\%\mathrm{GenX}\;\mathrm{removal}=\frac{C_{i}-C_{f}}{C_{i}}\times 100$$

where C_i_ and C_f_ are the initial and final GenX concentrations. For each sample, the adsorption test was performed three times and results were reported as an average ± standard deviation.

## 3. Results and Discussion

### 3.1. Morphology

**Figure 1:**
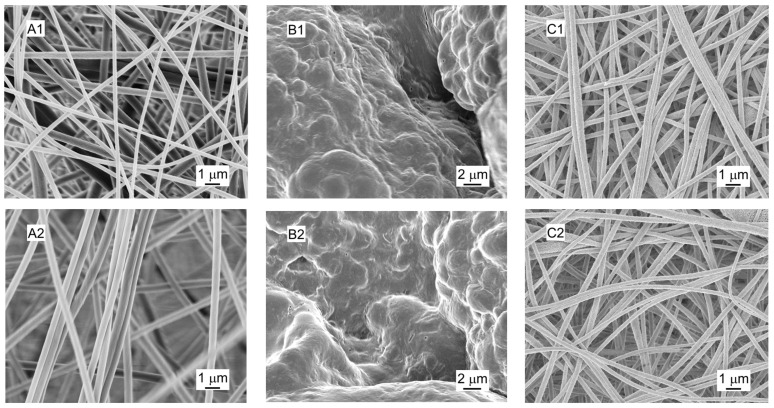
SEM images of the studied adsorbent materials after filtration of DI water (labeled as 1) and GenX water (labeled as 2) at pH 6: (**A**) ESPAN; (**B**) Algae; (**C**) ES(PAN/Algae).

The morphology of three adsorbent materials, i.e., the electrospun PAN/Algae (50/50) nanofibrous membrane (ES(PAN/Algae)) along with two controls: the electrospun PAN nanofibrous membrane (ESPAN) and the algae particles on electrospun PAN nanofibrous membrane (Algae), were characterized via SEM after filtration with DI water and 100 mg/L of GenX water (Figure 1). ESPAN showed a smooth surface with an average diameter of 522 ± 32 nm. ES(PAN/Algae) exhibited a rough surface with an average diameter of 295 ± 23 nm, which is approximately 43% smaller than that of the PAN nanofibers. Algae appeared as merged/agglomerated particles with sizes of 2 to 5 microns. After GenX adsorption, all the nanofibrous membranes retained their nanofibrous structure. The ESPAN nanofibers did not show any appreciable change in their diameter, but the average size of the ES(PAN/Algae) nanofibers increased ~28% to 378 ± 32 nm. In the case of the Algae, the particles shrunk a little bit, and their surface roughness was slightly reduced. Both the diameter changes in the ES(PAN/Algae) nanofibers and the morphology change on the surface of algae particles indicated an interaction between algae and GenX.

**Figure 2:**
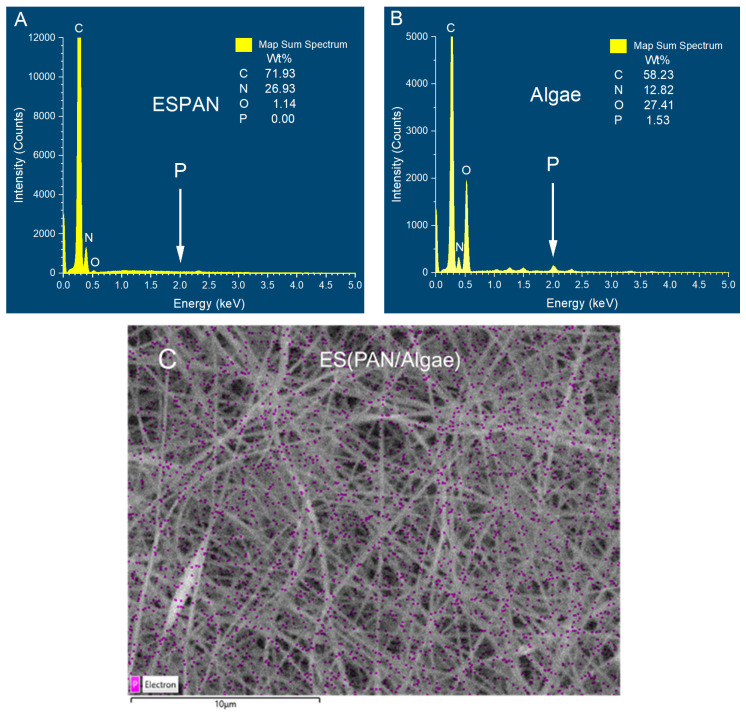
Elemental composition of ESPAN (**A**) and Algae (**B**) from EDX spectra with the phosphorus (P) element peak marked and P elemental mapping of the ES(PAN/Algae) (**C**) in which the P element is marked with magenta color.

To characterize algae distribution in the ES(PAN/Algae), we used phosphorus (P) elemental mapping through EDX. PAN does not contain any P element (Figure 2A) but P is an essential element in algae (Figure 2B). The P elemental mapping of the ES(PAN/Algae) nanofibrous membrane revealed that small algae pieces from raw algae particles due to sonication processing were indeed integrated with PAN in the electrospun nanofibers as submicrometer domains and distributed uniformly in the PAN matrix.

### 3.2. Surface Property

**Figure 3:**
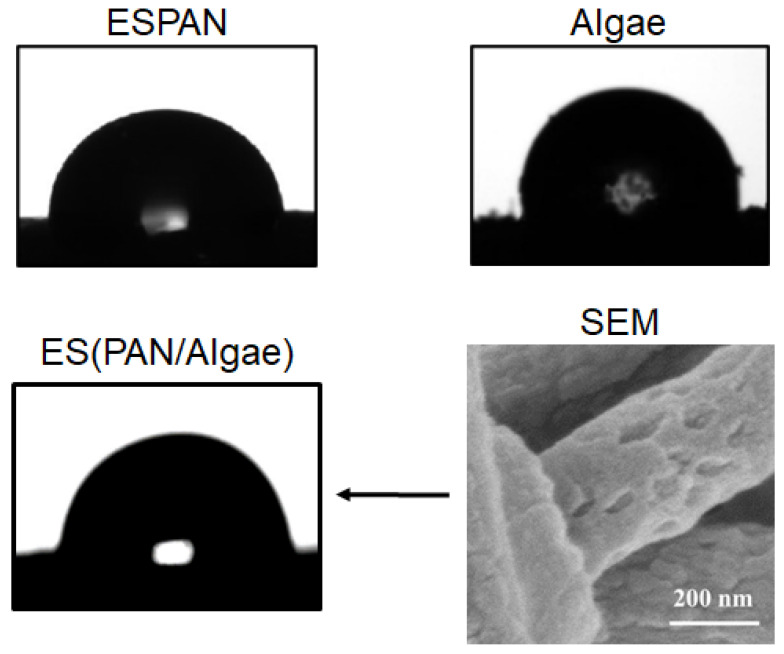
Optical images of water droplets on the ESPAN nanofibrous membrane, Algae particles, and the ES(PAN/Algae) nanofibrous membrane. The SEM image is a high-magnification image of the ES(PAN/Algae) nanofibers showing surface roughness.

To characterize surface property of the adsorbent/filter materials, water contact angle measurements were carried out and optical observations of water droplets on respective material surfaces are shown in Figure 3. It was observed that the ESPAN nanofibrous membrane was hydrophobic (water contact angle 103 ± 6.5°), which was in agreement with our previous research [20], while the Algae showed less hydrophobic surface properties (water contact angle 86 ± 1.4°). The ES(PAN/Algae) nanofibrous membrane exhibited less hydrophobicity (water contact angle 75 ± 1.4°). This smaller water contact angle could be attributed to the surface roughness of the ES(PAN/Algae) nanofibers as shown in the high-magnification SEM image in Figure 3, a result of the integration of small algae pieces (Figure 2C). The surface roughness enhanced water wettability according to the Wenzel equation.

### 3.3. Zeta Potential

**Figure 4:**
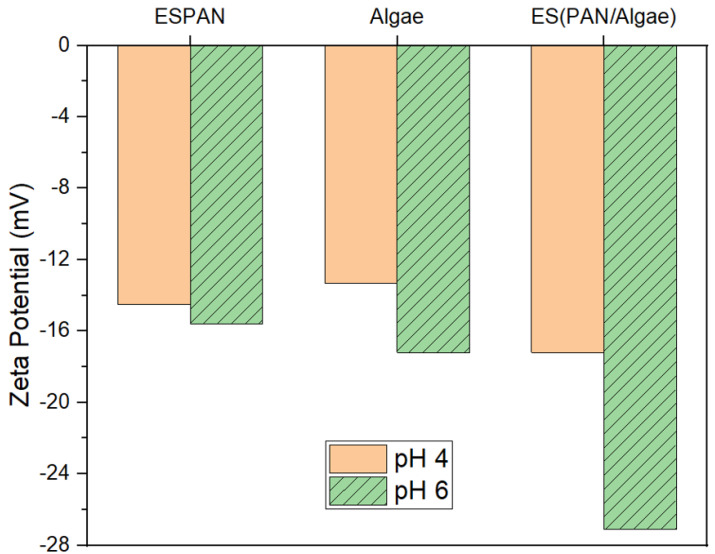
Zeta potential of ESPAN nanofibers, Algae particles, and ES(PAN/Algae) nanofibers at pH values 4 and 6.

Surface charges of the three adsorbent/filter materials were evaluated via DLS at pH values 4 and 6 (Figure 4). It was observed that all the tested materials possessed a negative charge irrespective of pH. The ESPAN nanofibers were negatively charged at pH 4 and became slightly more negatively charged with the increase of pH to 6, which is consistent with previous published research [21] and could be attributed to less protonation of the very polar nitrile functional groups from pH 4 to 6. Algae also possessed a negative charge due to the presence and balance of a large number of functional groups on the surface of algal cell walls, such as ionized carboxylic and amine functional groups [22]. With the increase of pH, the zeta potential of Algae became more negative, which is consistent with previous reports [23,24]. This can be ascribed to the increase in (-COO^−^) and decrease in (-NH_3_^+^) on algal surfaces at higher pH values. Compared to the ESPAN nanofibers and algae particles, the ES(PAN/Algae) nanofibers showed more negative charges at respective pH values and the same trend as the pH was increased. At pH 6, the most negative zeta potential (−27.1 mV) among all the studied adsorbent materials was observed in the ES(PAN/Algae) nanofibers. The sonification of algae in solvent as well as the electrospinning process should have broken the algal cell wall and exposed more negative-charged algal surface to water in the case of the ES(PAN/Algae) nanofibers, as confirmed by the P element mapping in Figure 2C.

### 3.4. GenX Remediation from Water

**Figure 5:**
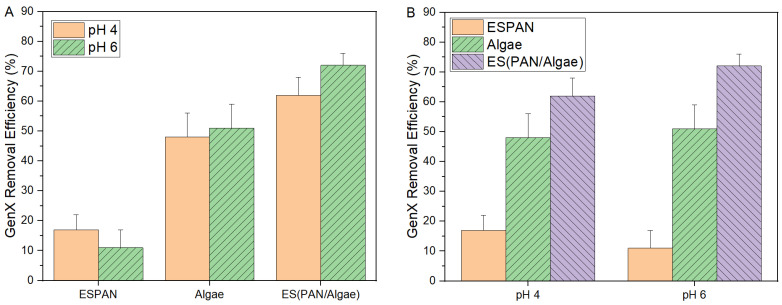
GenX removal performance of ESPAN, Algae, and ES(PAN/Algae) with material loading at 0.24 g/L: (**A**) pH effect on GenX removal efficiency of the adsorbent/filter materials; (**B**) material effect on GenX removal efficiency of the adsorbent/filter materials at pH values 4 and 6.

Quantitative adsorption of GenX on all the studied adsorbent/filter materials was carried out by using UV absorption [19] (Figure 5). A calibration curve of GenX was acquired in the concentration ranges of 10 to 100 mg/L at pH values 4 and 6, respectively, with a linear fitting coefficient of determination of R^2^ = 0.99.

The GenX removal efficiency of the ESPAN nanofibrous membrane at pH 4 is ~17%, which is approximately 50% higher than that at pH 6. Algae, i.e., the control sample with raw algae particles on top of the ESPAN membrane at a 50/50 weight ratio, showed a much higher GenX removal efficiency of ~50% regardless of pH. The maximum GenX removal efficiency from 100 mL of 100 mg/L GenX water was observed with the ES(PAN/Algae) nanofibrous membrane, which is ~72% at pH 6 while lower pH (pH 4) reduced GenX removal performance a little bit to ~62%. The 72% GenX removal efficiency indicated that the nanofibrous filter/adsorbent membrane reached its capacity with this filtration setup. To verify this, we tested the GenX removal efficiency of the ES(PAN/Algae) nanofibrous membrane with 200 mL of 100 mg/L GenX water and observed a reduced GenX removal efficiency of 38%, which is slightly higher than half of 72% from the 100 mL GenX water. This could be attributed to the longer residence time of GenX water in the funnel of the filtration setup, which increased possible contact time between GenX molecules and the ES(PAN/Algae) nanofibrous membrane to make it perform even closer to the ultimate GenX adsorption capacity. The observed maximum GenX removal capacity (weight-normalized GenX removal) was ~0.9 mmol/g, which is significantly higher than that of the ESPAN nanofibrous membrane and even the amidoxime surface-functionalized ESPAN nanofibrous membrane as shown in our previous research [19], as well as that of most published GenX adsorbents along with activated carbon [12]. It is noteworthy that the integration of algae in ESPAN nanofibers at 50 wt.% (0.012 g algae with 0.012 g PAN in the nanofibrous membrane) not only improved GenX removal from 100 mL of 100 mg/L GenX water at pH 6 by 6.5 times compared to that of the ESPAN nanofibrous membrane (0.024 g), but it also improved the GenX removal by 40% compared to the control Algae (0.012 g raw algae particles on top of 0.012 g ESPAN nanofibrous membrane), indicating an exciting synergistic effect from the integration of algae within PAN nanofibers. This could be attributed to the breakup of raw algae particles, and there was more algal surface exposed to water in the case of the ES(PAN/Algae) nanofibrous membrane.

### 3.5. GenX Removal Mechanism

The GenX removal mechanism from water using the ES(PAN/Algae) nanofibrous membrane was further studied via FTIR and XPS analysis. A pH value 6 was used in this part of the research because it was the pH of the GenX water and the ES(PAN/Algae) nanofibrous membrane showed the best adsorption performance at this pH.

#### 3.5.1. FTIR Analysis

**Figure 6:**
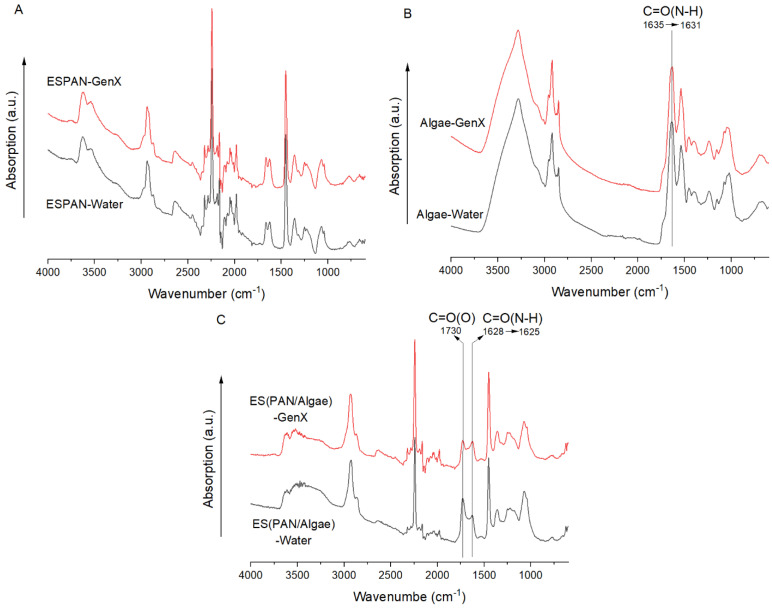
FTIR absorption spectra of ESPAN nanofibers (**A**), Algae (**B**), and ES(PAN/Algae) nanofibers (**C**) after filtration with DI water and GenX water, respectively, at pH 6. The filtration with water was used as a respective reference. The IR spectra were normalized by the C-H in the -CH_2_- of respective molecules at 1452–1454 cm^−1^.

FTIR was used to characterize the interaction between adsorbents and GenX (Figure 6). In GenX adsorption tests, respective control was obtained by testing the corresponding adsorbent with DI water (no GenX). There was no appreciable change in the FTIR spectra of the ESPAN nanofibrous membrane after GenX adsorption (Figure 6A). Algae particles showed an amide (I) (C=O(N-H)) band centered at 1635 cm^−1^ from their protein component and the amide band slightly shifted to 1631 cm^−1^ after GenX adsorption (Figure 6B), suggesting weakened C=O bonds due to the strong polar C-F bonds from the nearby GenX molecules.

Compared to algae alone, the algae in the ES(PAN/Algae) nanofibers showed a much-weakened amide I band, and the absorption peak shifted from 1635 cm^−1^ to 1628 cm^−1^. This indicated that the strong polar nitrile groups (C≡N) in PAN molecules interacted with the C=O groups in the amide (I) bonds in the algal protein component and resulted in a smaller dipole moment and force constant (Figure 6B,C). After GenX adsorption, the amide (I) band in the ES(PAN/Algae) nanofibers shifted from 1628 cm^−1^ to 1625 cm^−1^ (Figure 6C), exhibiting a similar C-F effect on C=O as shown in algae particles. It is noteworthy that the C=O in the ester bond (C=O(O)) was also weakened after GenX adsorption, suggesting that the strong polar C-F bonds from GenX molecules also interacted with the ester bonds in the lipid component of the algae.

#### 3.5.2. XPS Analysis

**Figure 7:**
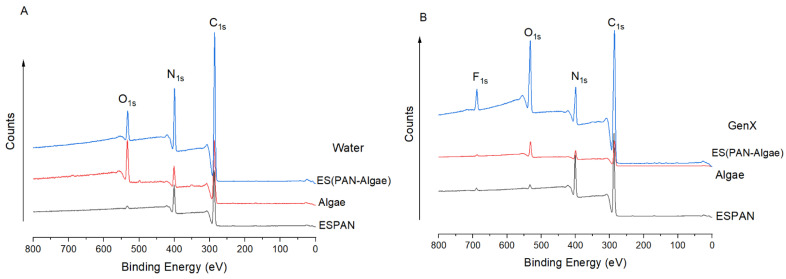
XPS spectra of all the adsorbents after filtration with water (**A**) and GenX water (**B**) at pH 6.

XPS was performed to analyze the surface elemental composition and bonding of the respective adsorbents after GenX adsorption (Figure 7). After the filtration of GenX water, the F_1s_ peak appeared on the XPS spectra of all the studied filter/adsorbent materials, confirming respective GenX adsorption.

To study the interaction between GenX and the respective adsorbents, high-resolution C_1s_ XPS spectra were used. The C_1s_ spectrum of the ESPAN nanofibrous membrane could be deconvoluted into three peaks, i.e., 284.8 eV, 286.46 eV, and 288.16 eV, corresponding to C-H/C-C, C≡N, and C=O, respectively [25,26]. After GenX adsorption, the C≡N peak shifted −0.22 eV and the C=O peak shifted −0.67 eV, respectively. The lower binding energy suggested that there is an interaction between GenX and PAN molecules. Specifically, the strong polar C-F bonds in GenX molecules (C(δ^+^)-F(δ^−^)) could form dipole–dipole interactions with the strong polar nitrile groups (C(δ^+^)≡N(δ^−^)), as well as with the strong polar carbonyl groups (C(δ^+^)=O(δ^−^)) in PAN molecules. These dipole–dipole interactions could increase the electron shielding effect on C_1s_ and thus reduce its binding energy. The high-resolution C_1s_ spectrum of algae particles could be deconvoluted into four major peaks, i.e., 284.8 eV, 286.36 eV, 287.67 eV, and 289.42 eV, corresponding to C-C/C-H, C-N/C-O, C=O, and O-C=O [27,28]. The binding energies of C-N, C=O, and O-C=O after GenX adsorption shifted towards the lower end, i.e., to 286.08 eV, 287.35 eV, and 288.49 eV, respectively, suggesting an interaction between algae particles and GenX due to the dipole–dipole interactions between these polar groups and the C-F bonds of GenX molecules, as well as possible hydrogen bonds between C-(N-H), C=O(N-H), and C=O(O-H) on the algal surface [22] and C=O/C-O-C in GenX molecules. These interactions increased the electron shielding effect on C_1s_ and reduced corresponding C_1s_ binding energies. The high-resolution C_1s_ spectrum of the ES(PAN/Algae) nanofibrous membrane could be deconvoluted into four major peaks, i.e., 284.8 eV, 285.84 eV, 286.93 eV, and 287.69 eV, corresponding to C-C/C-H, C≡N, C-N/C-O, and C=O, respectively. It is noteworthy that the binding energies of C≡N and C=O shifted −0.62 eV and −0.47 eV, respectively, compared to those of the ESPAN nanofibrous membrane, indicating an interaction between PAN and algae in the bicomponent nanofibrous membrane and specifically through dipole–dipole interactions between strong polar nitrile functional groups (C≡N) and algal surface functional groups (C=O). After GenX adsorption, the binding energy of C≡N further shifted −0.29 eV and the binding energy of C-N/C-O shifted −0.21 eV while the binding energy of C=O shifted +0.6 eV, all of which could be attributed to the attachment of GenX molecules to the adsorbent surface. The introduction of GenX molecules might have broken the previous dipole–dipole interactions between C=O and C≡N and resulted in a binding energy increase in C=O.

#### 3.5.3. Overall Discussion

The ES(PAN/Algae) nanofibrous membrane showed excellent performance in GenX adsorption at pH 6. At this pH, GenX molecules mostly exist with a negatively charged ionic end (-COO^−^) in water according to the pKa of GenX (3.82) and the Henderson–Hasselbalch equation [29,30]. The main GenX adsorption mechanism of the ES(PAN/Algae) nanofibrous membrane should not be caused by electrostatic interaction under this condition because of the repulsive force between the negative-charge-bearing GenX molecules (-COO^−^) and the negative surface charges of the nanofibers according to their large negative zeta potential (Figure 4). Based on the FTIR and XPS analysis, it is evident that the adsorbed GenX molecules have dipole–dipole interactions with the adsorbent material. Along with the water contact angle results, it is suggested that hydrophobic interaction should be the predominant adsorption mechanism for the ES(PAN/Algae) nanofibrous membrane. To verify this hypothesis, a GenX removal efficiency test was performed following the same procedure as described before but with NaCl in GenX water. It was demonstrated in a previous report [31] that the addition of NaCl to an aqueous solution could generate an electrostatic screening effect that significantly reduces adsorption capacity of the adsorbent if the adsorption is due to electrostatic force between the adsorbent surface and adsorbate ions. Specifically, 100 mL of 100 mg/L GenX water with 58.44 mg/L NaCl was used to test the GenX removal efficiency of the ES(PAN/Algae) nanofibrous membrane. The presence of NaCl, however, did not reduce the GenX removal efficiency of the ES(PAN/Algae) nanofibrous membrane, which confirmed that the adsorption of GenX by the ES(PAN/Algae) nanofibrous membrane is ascribed to non-coulombic interactions that could include hydrophobic interaction, dipole–dipole interaction, and hydrogen bonding. Due to hydrophobic interaction, the hydrophobic ends of GenX molecules could approach the ES(PAN/Algae) nanofibrous membrane followed by dipole–dipole interactions between C-F (GenX) and C≡N (PAN) and between C-F (GenX) and C=O (algae), as well as hydrogen bonding between C=O (GenX) and N-H/O-H (algae). Due to the homogeneous distribution of fine algal pieces in PAN nanofibers through sonication and electrospinning in the case of ES(PAN/Algae) nanofibrous membrane, there was more algal surface that could be exposed to GenX water during the filtration, which resulted in a synergistic effect for highly effective GenX remediation from water.

## 4. Conclusions

In summary, we demonstrated that *Chlorella* (the Algae) is an effective enhancer to be incorporated in electrospun PAN nanofibers to develop high-performance adsorbent/filter materials to remediate short-chain PFAS from water. We revealed an exciting synergistic effect for GenX, a representative short-chain PFAS, remediation from water by integrating 50 wt.% of *Chlorella* with PAN in the form of nanofibrous membrane through electrospinning (ES(PAN/Algae)), and observed a 72% GenX removal efficiency from 100 mL of 100 mg/L GenX water at pH 6 by using the prepared ES(PAN/Algae) as the filter through gravity filtration. The maximum GenX removal capacity of ES(PAN/Aglae) was ~0.9 mmol/g, which is significantly higher than that of the ESPAN nanofibrous membrane and other reported GenX adsorbents as well as activated carbon. This could be attributed to the homogeneous distribution of fine algal pieces in PAN nanofibers through sonication and electrospinning, which enabled much more algal surface to be exposed to adsorb GenX in water. The GenX adsorption of ES(PAN/Algae) could be mainly attributed to non-coulombic interactions including hydrophobic interaction, dipole–dipole interaction, and hydrogen bonding.

## Data Availability

The data presented in this study are available on request from the corresponding author.
